# Smooth Muscle Enfoldment Internal Sphincter Construction after Intersphincteric Resection for Rectal Cancer

**DOI:** 10.1371/journal.pone.0091491

**Published:** 2014-03-13

**Authors:** Heiying Jin, Bei Zhang, Hang Yao, Yonghong Du, Xiaofeng Wang, Qiang Leng

**Affiliations:** National center of colorectal surgery, the third affiliated Hospital of Nanjing University of Traditional Chinese Medicine, Nanjing, Jiangsu, China; University General Hospital of Heraklion and Laboratory of Tumor Cell Biology, School of Medicine, University of Crete, Greece

## Abstract

**Objective:**

To assess smooth muscle enfoldment and internal sphincter construction (SMESC) for improvement of continence after intersphincteric resection (ISR) for rectal cancer.

**Methods:**

Twenty-four Bama miniature pigs were randomly divided into a conventional ISR group and experimental SMESC group, with 12 pigs in each group. The proximal sigmoid colon was anastomosed directly to the anus in the ISR group. In the SMESC group, internal sphincter construction was performed. At 12 weeks before and after surgery, rectal resting pressure and anal canal length were assessed. Three-dimensional ultrasound was used to determine the thickness of the internal sphincter. After the animals were sacrificed, the rectum and anus were resected and pathological examinations were performed to evaluate the differences in sphincter thickness and muscle fibers.

**Results:**

All 24 animals in the SMESC group and the ISR group survived the surgery. Twelve weeks post-surgery, the rectal resting pressure, length of the anal high-pressure zone and the postoperative internal sphincter thickness for the ISR group were significantly lower than for the SMESC group. There was a thickened area (about 2 cm) above the anastomotic stoma among animals from the SMESC group; in addition, the smooth muscles were significantly enlarged and enfolded when compared to the ISR group.

**Conclusion:**

This animal model study shows that the SMESC procedure achieved acceptable reconstruction of the internal anal neo-sphincter (IAN/S), without increasing surgical risk. However, the findings in this experimental animal model must be confirmed by clinical trials to determine the safety and efficacy of this procedure in clinical practice.

## Introduction

Intersphincteric resection (ISR) plays an important role in sphincter-preserving surgery for patients with ultra-low rectal cancer [1], [2]. Martin et al. [3] carried out a systematic review of 14 studies on the ISR procedure performed for rectal cancers (1,289 cases) in which the overall 5-year survival and tumor-free survival rates were 86.3% and 78.6%, respectively; these findings indicate that the oncological outcome of the ISR procedure is satisfactory. In eight studies on anal function, the mean frequency of defecation per 24 hours, of patients, increased about 2.7 fold after the ISR procedure. In this regard, the functional outcome, after the ISR procedure in sphincter-preserving surgery among patients with low rectal cancers has not been optimal, although oncologically effective. In addition to the high defecation frequency, some patients reported a sense of urgency for defecation and leakage of stool, at night when asleep or otherwise not fully conscious. Chamlou et al. [4] reported that after the ISR procedure, approximately 41% of patients could control defecation, approximately 35% of patients had partial anal incontinence and 24% of patients had complete anal incontinence.

Various Factors, such as injury to the pelvic floor musculature, decrease of capacity in the rectum, shortening of the internal anal sphincter (IAS), may contribute to the decrease in the ability to control defecation after the ISR procedure [4]. In a study of the ISR procedure, in a pig model, Sato et al. [5] reported that the anal resting pressure was reduced to one third of its original value in the pigs undergoing the ISR procedure with complete excision of the internal anal sphincter. Therefore, some investigators recommend that the internal sphincter, on the side with the tumor, should be completely excised during the ISR procedure, and the opposite side, without a tumor, should be preserved. However, one third to one half of patients still have anal incontinence. In addition, whether inadequate excision of the lower edge of a tumor leads to an increase of local tumor recurrence is currently unknown.

Internal sphincter injury plays an important role in fecal incontinence following the IRS procedure. Reconstruction of an internal anal neo-sphincter (IAN/S) may be an effective way to improve anal continence postoperatively [6], [7]. The internal anal sphincter is the thickened portion of the rectal smooth muscles controlled by the splanchnic nerve, and has the ability to automatically contract and expand. During ISR reconstruction surgery, the colonic smooth muscles are able to automatically contract and expand, as controlled by the splanchnic nerve. Therefore, the question of whether the colonic smooth muscle could be enfolded and thickened to reconstruct an internal anal neo-sphincter (IAN/S) that could then be anastomosed to the anus was raised. The IAN/S would work as an IAS and enhance the anal resting pressure, alleviate the urgency to defecate, reduce the leakage of stool at night and improve the patient’s ability to control defecation. In this study, smooth muscle enfoldment and internal sphincter construction (SMESC), were studied in miniature pigs, to explore whether neo-sphincter plasticity could improve the ability to control defecation after rectal cancer surgery.

## Materials and Methods

### Animals

Twenty-four Bama miniature pigs (weight: about 10 kg, male or female) were used in this study (Animal license number: SYXK (su) 2010-0005). The Animal Ethics Committee of Jiangsu Academy of Agricultural Sciences approved this animal research.

At first, the pigs were anesthetized with ketamine and anal manometry was performed as well as 3D ultrasound examination of the internal and external sphincters. Anal manometry was used to assess the anal resting pressure and anal canal length. The 3D ultrasound was used to determine the thickness of the internal sphincter.

### Surgical Procedure

**Figure 1:**
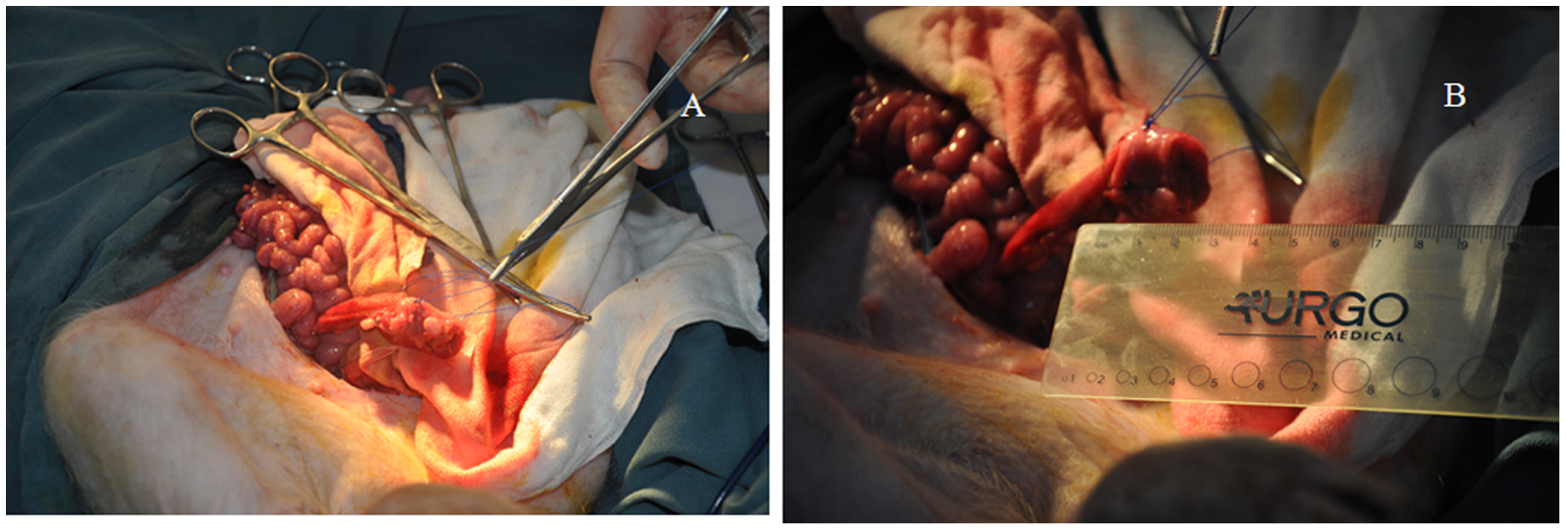
Surgical enfoldment process. A: Three sutures were placed along the longitudinal axis of the colon. B: After knots were tied, the 5 cm colon was shortened to 2 cm.

After intravenous anesthesia was provided, the rectum, to the level of levator ani muscle, was disassociated. A circular incision at the intersphincteric groove was performed and the internal sphincter was resected and disassociated upward to the abdominal wall. The rectum and part of the sigmoid colon at the site of the sigmoid colon were removed. The proximal sigmoid colon was directly anastomosed to the anus in the ISR group (5). In the SMESC group, at a distance of about 1 cm from the proximal end of the colon, a needle was inserted into the muscle layer of the colon wall, without penetrating the mucous layer. Continuous sutures were provided upward along the colon wall for about 5 cm, with a needle spacing of about 0.5 cm. Then, the needle was withdrawn. After completing the sutures, on both sides, and the rear side using the same method, the stitches were tightened to shorten the 5 cm colon wall to 2 cm, and then they were knotted; finally, the colon was made to prolapse outside of the anus to be anastomosed. The abdominal incision was closed, and the operation was completed (**Figure 1a,b**).

### Postoperative Functional Evaluation

Anorectal manometry and 3D ultrasound examination were carried out 12 weeks post-surgery. Anorectal manometry was used to assess the rectal resting pressure and anal canal length. The 3D ultrasound was employed to determine the thickness of the internal sphincter. After anorectal manometry and 3D ultrasound examination, the animals were sacrificed and the rectum and anus were resected. Pathology examinations were performed to compare the differences in sphincter thickness and muscle fibers between the ISR group and the SMESC group.

### Statistical Analysis

The data are presented as the mean and S.E.M. and analyzed using the student *t*-test. A P<0.05 was accepted as significant.

## Results

In the experimental group (the SMESC group) and the control group (ISR group), were compared postoperatively. In the ISR Group, 12 animals had fecal stains that were observed on their tails within two weeks post surgery. Formed stool was noted at two weeks after the operation. In the SMESC group, 12 animals had fecal stains that were observed on their tails within two weeks after the operation and 11 animals could defecate normally by two weeks after the operation. One animal had difficulty with defecation due to anal stenosis. It could defecate after the anus was dilated under local anesthesia.

With regard to anorectal manometry, there was no significant difference in the preoperative rectal resting pressure, length of the anal high-pressure zone and thickness of the internal sphincter among the Bama pigs in the two groups. However, 12 weeks post-surgery, the rectal resting pressure was reduced to 9.2 cmH_2_O (29.4%) in the ISR group, and was 23.7 cmH_2_O (79.5%) in the SMESC group; this difference was statistically significant. With regard to the anal high-pressure zone, the length of the anal high-pressure zone was reduced to 8.4 mm (41.4%) in the ISR group, and was 16.7 mm (74.6%) in the SMESC group; this difference was statistically significant. The 3D ultrasound showed that the internal sphincter thickness was decreased to 0.9 mm (40.9%) in ISR group, postoperatively, and was 1.7 mm (85.4%) in the SMESC group; this difference was statistically significant. A comparison of the changes in anorectal manometry and 3D ultrasound examination between the ISR and SMESC groups is shown in **Table 1**.

**Table 1:** Comparison of changes in anorectal manometry and 3D ultrasound between the ISR and SMESC groups (n = 24).

	Pre-surgery			Twelve weeks post-surgery		
	ISR	SMESC	P value	ISR	SMESC	P value
Rectal resting pressure (cm H_2_O)	31.2±6.1	29.8±5.9	0.874	9.2±1.4	23.7±3.4	0.004
Length of anal high-pressure zone (mm)	20.3±2.4	22.4±1.7	0.935	8.4±1.5	16.7±3.2	0.007
Thickness of internal sphincter (mm)	2.2±0.2	1.99±0.3	0.826	0.9±0.4	1.7±0.9	0.037

**Figure 2:**
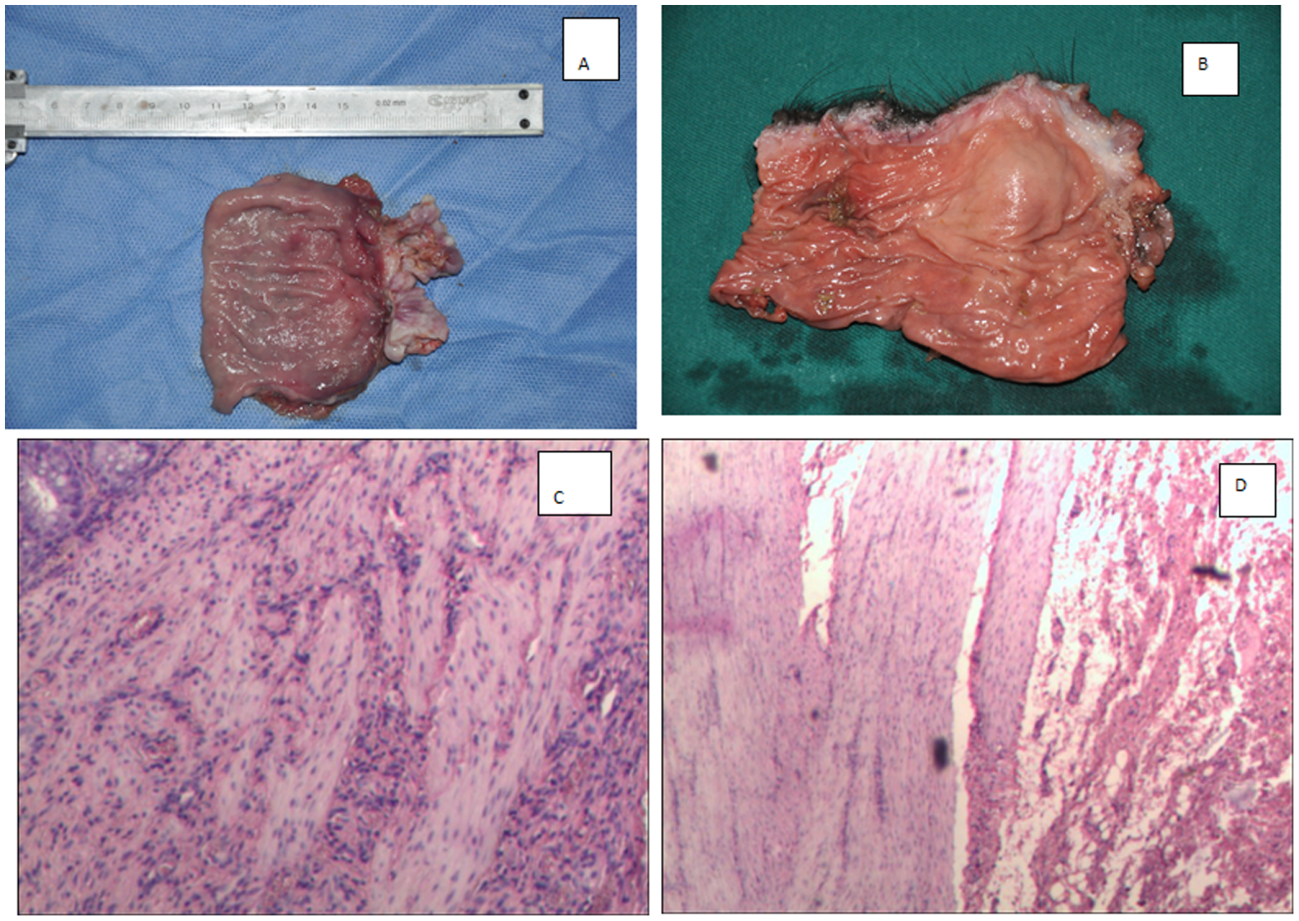
Postoperative specimens from the SMESC and ISR groups and results of pathological examinations. A: The area above the anastomotic stoma was observed to be thickened in specimens from the SMESC group. B: There was no thickened area in the specimens from the ISR group. C: In the SMESC group, the smooth muscles were enfolded and thickened under the light microscope at a magnification of x200. D: In the ISR group, the smooth muscles were not enfolded or thickened under the light microscope at a magnification of x200.

Per naked eye observation, there was a thickened area, about 2 cm above the anastomotic stoma among animals in the SMESC group postoperatively. By contrast, there was no thickened area in the animals without enfoldment in the ISR group. The pathology showed that the smooth muscles were significantly enlarged and enfolded in the SMESC group, but not in the ISR group (Figures 2a,b,c,d).

## Discussion

Although there are a variety of factors that contribute to fecal incontinence, injury to the internal sphincter muscle plays an important role in the fecal incontinence that occurs after the ISR procedure [6], [7]. Sato et al. [5] reported that the anal resting pressure was reduced to one-third of its original value in pigs undergoing the ISR procedure with complete excision of the internal anal sphincter. Other studies have also indicated that after the ISR procedure, the lack of an internal sphincter is an important cause of decreased anal function [8], [9], [10]. The internal sphincter is formed by a thickening of the circular smooth muscles of the rectum; can the smooth muscles be thickened artificially by enfolding the rectal smooth muscles to reconstruct an internal anal neo-sphincter (IAN/S) and can this construct effectively replace the function of the internal anal sphincter?

To answer these questions, a pig model of smooth muscle enfoldment internal sphincter construction (SMESC) was performed to reconstruct an IAN/S. The sphincter was thickened 2.5-fold. The surgery did not increase complications following the ISR procedure. All pigs had good anastomotic healing and no leakage or abscess formation after the operation. In the SMESC group (smooth muscle enfoldment group), one pig had anastomotic stenosis, which improved after anal dilation under local anesthesia. The results of this study show that the SMESC did not increase surgical complications when compared to the ISR group.

With regard to postoperative function, the animals in both groups could defecate normally after the surgery. Although the phenomena of postoperative defecation and fecal stains observed on the tails of pigs were studied by Sato et al [5], in this study, active defecation was not considered as an outcome measure. The major reason for this is that the defecation process of pigs is not fully understood. Use of postoperative defecation and fecal stains on the tails of the pigs to assess postoperative anal function, is too subjective. In this study the anal resting pressure, the length of the anal high-pressure zone and the thickness of the internal sphincter were used to assess anal function after the ISR and SMESC in the pigs. The results of this study showed that the postoperative anal resting pressure in the SMESC group was about 2-fold higher than that of the ISR group; these findings suggest that the SMESC improved the anal resting pressure in the pigs. Since more than 80% of anal resting pressure is generated from the internal anal sphincter, the enfolded smooth muscles functioned partially as an internal sphincter [11], [12]. With regard to the anal high-pressure zone, the value measured in the SMESC group was nearly two-fold higher than in the ISR group. These findings further confirmed that the enfolded smooth muscles played an important role in the internal sphincter function. For the thickness of the internal sphincter, it was reduced to 40.9% after surgery in the ISR group, but only reduced to 85.4% in the SMESC group. The significant difference between the two groups confirmed that the smooth muscles could be enfolded and thickened to form a structure similar to the internal sphincter. Based on the results of naked eye observation and pathological examination, after surgery, compared to the specimens from the ISR group, the specimens in the SMESC group had thickened muscles in the area enfolded, and the smooth muscle fibers were noted to be thickened, with the morphological characteristics of an internal sphincter [13], [14]. The results of this study showed that the enfolded bowel smooth muscle that was constructed caused thickening of the internal anal neo-sphincter around the anus and the reconstructed anal high pressure zone, which may work as a passive barrier at rest and improve ano-rectal function in patients with otherwise compromised pelvic floor musculature.

Nevertheless, whether the enfoldment of smooth muscles will cause a decrease in the compliance of a newly built rectum was not evaluated in this study; the examination of rectal compliance requires the cooperation of human subjects. It is difficult to obtain these data from animal experiments [8].

The results of this study suggest that the SMESC may improve anal function after the ISR procedure. This result is preliminary based on an animal model. Whether the findings are the same in humans requires further study in clinical trials. A pilot study is planned to confirm the safety and efficacy of this procedure in humans.

In summary, the results of this animal model experiment showed that the SMESC increased anal resting pressure, the length of the anal high-pressure zone and the thickness of the anal sphincter. These findings imply that the SMESC can play an important role in reconstruction of the internal anal neo-sphincter (IAN/S), without increasing surgical risk.
